# Antibody Response to Rotavirus C Pre-Farrow Natural Planned Exposure to Gilts and Their Piglets

**DOI:** 10.3390/v14102250

**Published:** 2022-10-14

**Authors:** Deepak Kumar, Amanda V. Anderson, Jeremy Pittman, Nora L. Springer, Douglas G. Marthaler, Waithaka Mwangi

**Affiliations:** 1 Department of Diagnostic Medicine/Pathobiology, College of Veterinary Medicine, Kansas State University, Manhattan, KS 66506, USA; 2 Department of Veterinary Diagnostic and Production Animal Medicine, College of Veterinary Medicine, Iowa State University, Ames, IA 50011, USA; 3 Smithfield Foods, Inc., 434 E Main St., Waverly, VA 23890, USA; 4 Clinical Pathology, Biomedical and Diagnostic Sciences, College of Veterinary Medicine, University of Tennessee, Knoxville, TN 37996, USA; 5 Indical Inc., 1317 Edgewater Dr #3722, Orlando, FL 32804, USA

**Keywords:** swine, rotavirus C, natural planned exposure, ELISA, sequencing

## Abstract

A longitudinal study was conducted to investigate the dynamics of genotype-specific (G6 and P[5]) antibody response to different doses (3, 2 and 1) of rotavirus C (RVC) natural planned exposure (NPE) in gilt serum, colostrum/milk and piglet serum, and compare with antibody response to rotavirus A NPE (RVA genotypes G4, G5, P[7] and P[23]). G6 and P[5] antigens of RVC were expressed in mammalian and bacterial cells, and used to develop individual indirect ELISAs. For both antigens, group 1 with 3 doses of NPE resulted in significantly higher IgG and IgA levels in colostrum compared to other groups. In piglet serum, group 1 P[5] IgG levels were significantly higher than other study groups at day 0 and 7. Piglet serum had higher IgA levels for group 1 piglets compared to other groups for both antigens. A comparison of colostrum antibody levels to rotavirus A (RVA) and RVC revealed that colostrum RVC IgG and IgA titers were lower than RVA titers irrespective of the G and P-type. Next generation sequencing (NGS) detected same RVC genotypes (G6 and P[5]) circulating in the piglet population under the window of lactogenic immunity. We conclude that the low RVC load in NPE material (real-time PCR Ct-values 32.55, 29.32 and 30.30) failed to induce sufficient maternal immunity in gilts (low colostrum RVC antibody levels) and passively prevent piglets from natural RVC infection in the farrowing room. To the best of our knowledge, this is the first study comparing differences in antibody response to porcine RVA and RVC in a commercial setting.

## 1. Introduction

Rotaviruses (RVs) are non-enveloped viruses in the Rotavirus genus of Reoviridae family. Eleven segments of double-stranded RNA encoding six structural proteins (VP1, VP2, VP3, VP4, VP6 and VP7) and 5 non-structural proteins (NSP1–NSP5/6) are encoded in a 18 kb RV virion [1,2]. VP7, a glycoprotein with a molecular weight of 37 kDa, constitutes 30% of the virus protein, and forms the smooth external surface of the outer shell. The minor component of the outer shell, VP4, is present as a series of spikes that project outward from the VP7 shell. VP4 is non-glycosylated, has a molecular weight of 88 kDa, and constitutes 1.5% of the virus protein [3]. The VP4 gets proteolytically cleaved into VP5 and VP8. The VP8* forms the spike of the virion and assist in host attachment and infectivity [4,5]. Both VP7 and VP4 proteins independently induce neutralizing and protective antibodies [6,7].

Currently, ten RV species, A through J (RVA-RVJ) have been classified on the basis of sequencing of the VP6 gene [8,9]. A binary classification system is used to address vast rotavirus diversity on the basis of sequencing of G (VP7) and P types (VP4). The dual (G/P) tying system has been extended to a complete genome classification system based on nucleotide sequencing of all 11 RV segments with nucleotide percent identity cut-off values set for each segment. Out of 10 RV species, only species A, B, C, E, and H have been reported from swine [10,11,12,13]. RVC has been detected from a variety of sources including swine [14,15,16], humans [17,18], cows [19], ferrets [20], cats [21], and dogs [22]. First detected in 1980, porcine RVC was considered to have a moderate prevalence rate (4–31%) [23]. Previously, RVA was considered the more prevalent and pathogenic in pigs, however recent data suggest that RVC is a major cause of diarrhea in neonatal pigs, particularly in piglets younger than 3 days old [24,25]. The G6 genotype (70%) is the dominant RVC genotype followed by G5 (17%), G1 (12%), and G9 (1%). A prevalence as high as 76.1% has been reported from the piglet population in the US [26].

There is no in utero transfer of antibodies in swine due to epitheliochorial placenta. Hence, piglets are completely dependent on colostrum intake of maternal derived antibodies in colostrum and milk [27]. IgG and IgA produced in the sow traffics to the mammary glands and transferred through colostrum and milk to the piglets, where RVs are locally neutralized in the gut [26,28,29,30]. IgG is the most common immunoglobulin found in swine colostrum and provides against systemic infections. Secretory IgA (sIgA) is more prevalent in milk and is associated with the protection conferred at mucosal level [27,31].

High prevalence of rotavirus C (RVC) in neonatal piglets is a major concern to swine producers. Epidemiological data suggest that RVC infections are more prevalent among neonatal piglets than weaned piglets, however the reasons are not completely understood [15,26]. Likely reasons include lack of RVC vaccine for use in swine, insufficient maternal RVC antibodies in colostrum, low minimum infectious dose of RVC required for infecting piglets compared to other swine enteric viruses, and a distinct pathogenic mechanism compared to RVA [26].

Despite being the most common cause of RV diarrhea in piglets less than 1 weeks of age, only one vaccine is available against RVC due to the inability of RVC to adapt to the cell culture. A vectored virus vaccine platform known as Sequivity has been introduced by Merck animal health for use in pre-farrow gilts/sows against RVs. However, its field efficacy data is not available to assess the actual protection conferred to the swine against RVCs. Hence, natural planned exposure (NPE) to gilts prior and during pregnancy is the most widely used method to stimulate lactogenic immunity against RVs in the US swine herds [32,33]. RV-infected material from feces or intestines of sick piglets is fed to pregnant gilts and sows to boost maternal immunity against RVs. Studies investigating the efficacy of NPE protocols against RVC in swine are completely lacking. Moreover, there is a lack of commercial ELISAs for swine RVC, which hampers effective assessment of NPE protocols. There is only one report of RVC virus like particle (VLP) based ELISA to detect genotype-specific RVC antibodies in gilt/sow serum and lacteal secretions [26]. Hence, this study was carried out to investigate the maternal immunity induced by pre-farrow RV NPE to gilts and passive lactogenic immunity to piglets using in-house developed RVC genotype-specific indirect ELISAs.

## 2. Materials and Methods

### 2.1. Study Design, NPE Material and Sampling

The study was conducted on an 1800-head commercial, breed-to-wean gilt farm in the United States. Pregnant gilts were randomly allocated into 4 study groups. Group 1 received 3 doses of NPE at 5, 4, and 3 weeks pre-farrow (WPF), group 2 received 2 doses of NPE at 5 and 3 WPF, group 3 received one dose of NPE at 5 WPF, and group 4 received no NPE (control group) (Table 1). Gilts were housed by treatment group, and all movement between groups was prohibited. Forty-six litters (Group 1 = 12, Group 2 = 12, Group 3 = 11, Group 4 = 11) were evaluated for rotaviral fecal shedding and antibody titers. 

A real-time PCR based surveillance was performed on the sow farm prior to the study and the NPE material was created according to the prevalent RVA and RVC strains on the farm. NPE material fed to the gilts contained both RVA and RVC strains. Details of gilts and piglet’s antibody response to RVA in the NPE material have been described elsewhere [34]. NPE material was created using the master seed method and stored in an on-farm deep freezer [32]. RV-infected material collected at the gilt multiplier site was used to inoculate colostrum-deprived piglets (n = 3). After 24 h, the piglets were euthanized and their intestinal contents were processed to create NPE material. To prepare NPE material, 40 mL of the intestinal content material (master seed) was mixed with approximately 14 L of water and enough feed to generate 100 doses (cups) of gruel mixture. Each dose of NPE contained approximately 237 mL of intestinal content material and each gilt received 1 dose of NPE gruel, administered 5 h after daily feeding. A sample of each NPE material was collected for real-time PCR testing and sequencing.

Blood samples from gilts were collected at weeks −5, −3, 0 (farrowing) and 3. Colostrum was collected at birth and milk was collected 1–3 weeks post farrowing. Five piglets from each litter were selected for serum sample collection throughout the study. Blood samples from 5 piglets per litter were collected at weeks 0 (farrowing), 1, 2, 3, 4, 5, and 6 for a total of 7 blood samples per piglet. No intra-litter movement of pigs was allowed. Furthermore, care was taken to include healthy and visually similar weighed piglets to maintain uniformity in piglet sizes [29].

### 2.2. Generation of Rotavirus C VP7 and VP4* Expression Constructs

NGS identified G6 and P[5] RVC genotypes in the NPE material. G6 VP7 was expressed using mammalian Expi293TM Expression System (Gibco) and P[5] VP4* was expressed using bacterial expression system. VP7 sequence of G6 RVC was modified to add in-frame 8-his tag and streptavidin tags at N and C terminals, respectively, to track protein expression and affinity purification of recombinant proteins. Gene sequences was codon optimized for mammalian expression. A kozak sequence was added at N terminal to facilitate enhanced protein expression. Linker sequences were added just preceding each affinity tag. CD5 secretory signal was fused at N-terminal for efficient secretion of the recombinant protein into the culture media. The synthetic rotavirus VP7 genes were subcloned into pcDNA3.1+ mammalian expression vector (InvitrogenTM). Truncated VP4* (aa26-476) of P[5] RVC was cloned in to pET-24a(+) vector with a linker followed by a 8-his tag at C-terminal. Codon optimization, gene synthesis, cloning into pcDNA3.1 (+) and pET-24a(+) vectors, and gene sequence validation were outsourced to Genscript.

### 2.3. Recombinant Protein Expression, Purification and Validation

G6 VP7 pcDNA3.1 (+) plasmid construct was transformed into DH5α competent cells and positive clones were used for recombinant protein expression in the mammalian Expi293TM Expression System (Gibco). For bacterial expression, pET-24a(+) plasmid carrying P[5] VP4* was transformed into Rosetta cells (Thermo Fisher Scientific) and grown overnight on LB agar plates with 30 µg/mL kanamycin at 37 ℃. Individual colonies were amplified overnight in 20 mL of LB broth with kanamycin at 37 ℃ with overnight shaking. The overnight culture was added to 1 L of LB broth with kanamycin (30 µg/mL), grown at 37 ℃ with shaking until reaching an OD600 approximately 1. Cultures were induced with IPTG added to a final concentration of 0.5 mM for 16 h at 16 ℃ with shaking. After expression, bacterial cultures were centrifuged and the resulting cell pellets were used for protein purification. Recombinant proteins were purified using immobilized metal affinity chromatography (IMAC) using TALON Cobalt resin (Takara Bio, San Jose, CA, USA) following a hybrid batch/gravity procedure provided by the manufacturer with some modifications. The affinity purified proteins were quality control validated by Western blotting and pure protein fractions were pooled and concentrated using 10 K protein concentrators. Contentrated proteins were quantified using BCA assay and stored at −80 °C until further use.

### 2.4. Development of Recombinant Protein ELISAs to Quantitate RVC Antibodies

Indirect ELISAs for G6 and P[5] RVC were optimized to detect genotype-specific RVC IgG and IgA antibodies in porcine serum and colostrum/milk. A checkerboard titration method was used to determine optimal coating antigen concentration and secondary antibody concentrations (anti-IgG and anti-IgA). Other ELISA parameters such blocking conditions, ELISA plate incubation time and temperature, and washing steps were also individually optimized. The ELISA antibody titer was expressed as the reciprocal of the highest dilution that had a A410 value greater than twice the mean of negative control wells.

### 2.5. Screening Serum and Milk of Gilts and Piglet Serum for RVC Antibodies Using Genotype-Specific ELISA

Blood samples were centrifuged at 2000× *g* for 15 min to obtain serum and stored at −80 °C until use. Colostrum and milk samples were centrifuged at 5000× *g* overnight at 4C to separate fat, debris and whey. Fat layer was carefully separated using sterile pipette tips and clear fluid (whey) was collected in sterile 2 mL Eppendorf tubes. Whey was stored in minus 80 until further use. To determine endpoint titer of RVC IgG and IgA antibodies, serum and colostrum/milk samples were serially diluted (1:200, 1:400, 1:800, 1:1600, 1:3200, 1:6400, 1:12,800 and 1:25,600) in 5% NFDM prepared in 1× PBST and added (100 µL) in duplicates to the wells of overnight protein coated, blocked and washed immunoassay plates. Anti-porcine IgG (1:10,000 in 5% NFDM, 100 µL) and IgA (1:3000 diluted in 5% NFDM 1× PBST, 100 µL) conjugated to horseradish peroxidase (Abcam, Cambridge, UK) was added to each well and incubated at 37 °C for 1hour. The end point titer was expressed as the reciprocal of the highest dilution that had a A410 value greater than twice the mean of negative control wells. If any sample had antibody titer more than the higher end of dilution range (>1:25,600), that sample was retested with more dilutions (1:25,600–1:102,400). Each ELISA plate contained a serially diluted positive and negative control to avoid plate to plate variation. Since, true positive controls (antiserum against each protein) were not available, few high titer serum samples were pooled and used as positive control throughout the ELISA testing to maintain uniformity in sample testing.

### 2.6. Next Generation Sequencing and Real-Time PCR to Detect RVC in NPE Material and Piglet Feces

Whole genome sequencing (WGS) of RVC strains in the NPE material were to be compared with the RVC sequences recovered from piglet feces to characterize the genetic differences between NPE and RVC strains shed by piglets. NPE material was sequenced at the beginning of the animal study by NGS at Molecular NGS laboratory at Kansas State Veterinary Diagnostic Laboratory (KSVDL), Kansas State University. Direct-zol RNA Miniprep kit (Zymo Research, Irvine, CA, USA) was used to extract Rotavirus dsRNA from the NPE material. We employed a single primer amplification technique (SPAT) protocol to amplify cDNA sequences from dsRNA [35]). A P1 primer (5′Phos/CCGTCGACGAATTCTTT/3′AmMO) was annealed to the dsRNA and extracted. First strand synthesis was carried out using a SuperScriptTM III First-Strand Synthesis (ThermoFisher Scientific, Waltham, MA, USA) and the P2 primer complementary to P1 (5′-AAAGAATTCGTCGACGGG-3′). The cDNA was amplified using an LA Taq DNA polymerase kit (Takara Bio USA, Mountain View, CA, USA), and the PCR products were purified using QIAQuick PCR Purification Kit (Qiagen, Germantown, MD, USA). NGS was performed on the Miseq (Illumina, San Diego, CA, USA) platform. Raw reads were trimmed and assembled using de novo and reference-based assembly using CLC Genomics Workbench (CLC Bio, Redwood City, CA).

A multiplex semi-quantitative real-time PCR (RT-PCR) assay was used for the detection of RVA and RVC in NPE material and fecal samples, and reported as cycle threshold (Ct) values [15]. A Ct value cut off of 36 was used to declare a sample negative for RVC. Piglet fecal samples were chosen for sequencing from weeks 0–3 samples to assess viruses shed in the presence of lactogenic immunity. Litters from which RVC was detected for multiple weeks in a row with Ct values less than 26 (high RVC load) were selected for sequencing. A total of 30 piglet fecal samples from all 4 groups were submitted for sequencing. Whole genome sequencing (WGS) of piglet fecal samples was outsourced to the Centers for Disease Control and Prevention (CDC), Atlanta, Georgia.

### 2.7. Statistical Analysis

The significance of the differences between the treatment and the control groups was determined by two-way Analysis of Variance (ANOVA). Statistical analysis was performed using GraphPad Prism 7 (Version 7.04, GraphPad Software, Inc., La Jolla, CA, USA) and a significance level of *p* < 0.05 was used for all analyses.

## 3. Results

### 3.1. Recombinant Protein Expression

**Figure 1:**
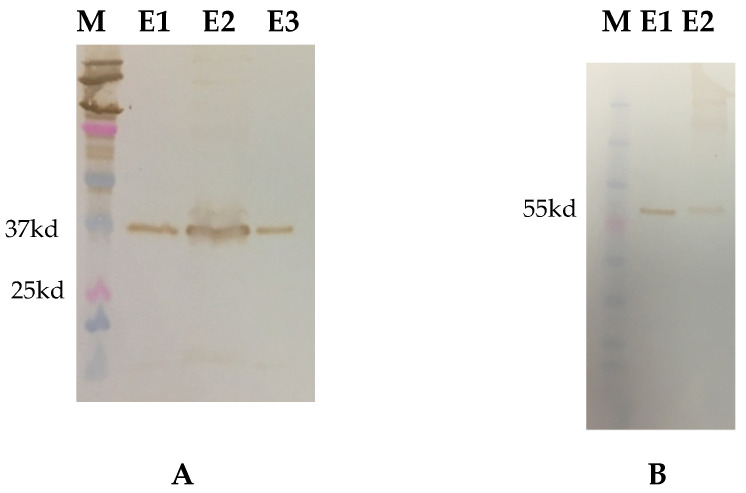
Western blot confirmation of affinity purified proteins. (**A**) Purified G6 RVC VP7 (37 kd), (**B**) Purified P[5] VP4* (55 kd), M—Protein marker, E1, E2 and E3—protein elutes.

**Figure 2:**
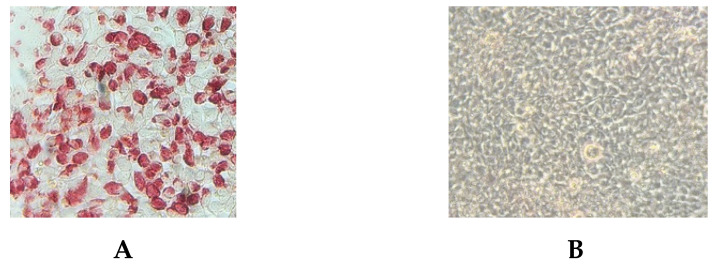
Protein expression by the constructs encoding RVC VP7 was evaluated by immunocytometric analysis of HEK 293A cells. (**A**) Cells transfected with pcDNA3 constructs encoding G6 VP7 of rotavirus C. Transfected cells were probed with anti-his monoclonal antibody. (**B**) Negative control.

G6 antigen was efficiently secreted into the Expi293 culture media. An estimated 37-kDa and 55-kDa bands corresponding to the expected molecular weight of recombinant VP7 and truncated VP4* antigens were detected on Western Blot using anti-His monoclonal antibodies (Figure 1). Expression levels and yields of VP4* antigen were low compared to VP7. Immunocytometric analysis of HEK-293A cells transfected with pcDNA3.1 (+) plasmid carrying G6 gene and probed with anti-his monoclonal antibodies confirmed antigen expression (Figure 2). Coating concentrations of 50 ng/well for both antigens resulted in optimal ELISA OD value readouts. Blocking the ELISA plates with 5% NFDM prepared in 1× PBST with 0.05% Tween-20 and four washings after each incubation step resulted in minimal background. The optimal incubation temperature and time combination for samples (serum/colostrum/milk) and secondary antibodies was at 37 ℃ for 1 h. Concentrations of 1:10,000 and 1:3000 for peroxidase conjugated IgG and IgA were found optimal.

### 3.2. Antibody Response to RVC NPE

#### 3.2.1. Gilt Serum

**Figure 3:**
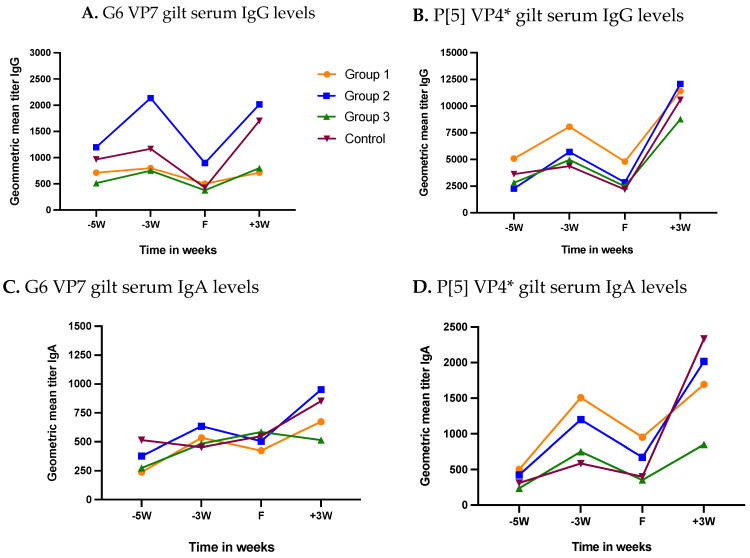
Kinetics of longitudinal gilt serum antibody response to RVC NPE. Progression of RVC IgG and IgA levels over time in gilts receiving three (group 1), two (group 2), one (group 3) or no (group 4) doses of natural planned exposure. Gilts farrowed at week 0 (F). (**A**) G6 VP7 IgG levels, (**B**) P[5] VP4* IgG levels, (**C**) G6 VP7 IgA levels, (**D**) P[5] VP4* IgA levels. Horizontal axis represents multiple sample collection time-points (−5W = 5 weeks pre-farrow; −3W = 3 weeks pre-farrow; F = at farrowing; +3W = 3 weeks post-farrow or at weaning). Vertical axis represents geometric mean antibody titers for respective study groups.

All gilts irrespective of the study group had some levels of anti-RVC antibodies before the administration of 1st NPE dose at 5-weeks pre-farrow (WPF) (Figure 3A–D). Geometric mean titers (GMT) IgG levels at 5 WPF for G6 and P[5] genotypes were in the range of 514.67–1198.55 and 2262.74–5079.68, respectively (Figure 3A,B). Two doses of NPE in group 1 (5 and 4 WPF) and one dose each in group 2 and 3 (5 WPF) resulted in elevated IgG levels at 3 WPF for both G6 and P[5]. Control group IgG levels at 3 WPF showed a minimal increase in the absence of NPE. Serum IgG levels dropped sharply in all treatment groups at farrowing (F) followed by a quick rebound until 3-weeks post-farrowing (weaning) for both antigens. P[5] IgG GMTs at 3 WPF in all treatment groups were at least 5-fold higher than G6 IgG levels (Figure 3A,B).

GMT IgA levels at 5 WPF were G6 (GMT 237.44–514.67) and P[5] (GMT 236.77–503.97) (Figure 3C,D). Gilt serum IgA levels for both proteins in treatment groups increased at 3 WPF after respective NPE doses. Control group gilt serum IgA levels for G6 decreased at 3 WPF and showed a slight increase for P[5] at 3 WPF (Figure 3C,D). Similar to IgG levels, IgA levels also increased sharply post-farrow until weaning.

#### 3.2.2. Colostrum and Milk

**Figure 4:**
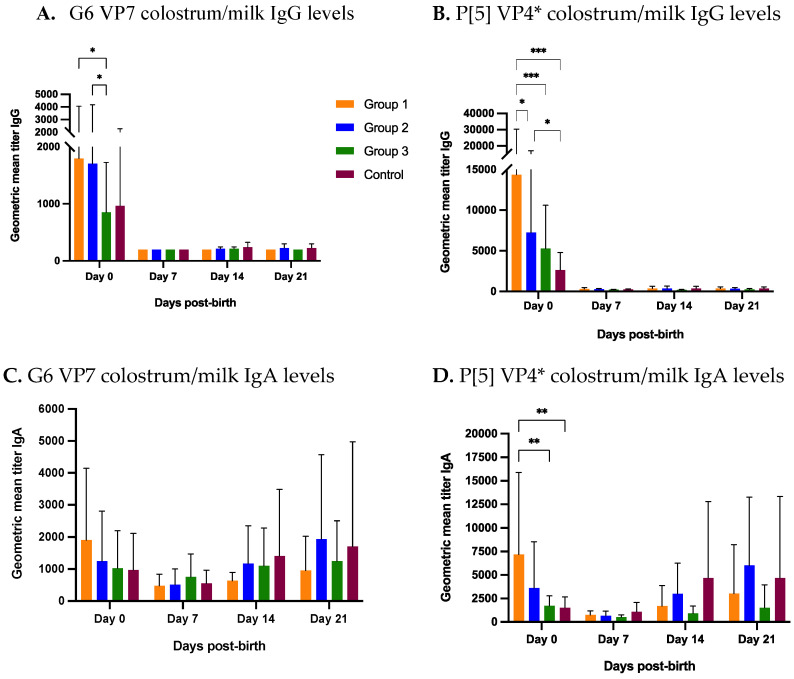
Longitudinal colostrum/milk antibody levels against RVC. Progression of RVC IgG and IgA levels over time in gilts colostrum/milk receiving three (group 1), two (group 2), one (group 3) or no (group 4) doses of natural planned exposure. Gilts farrowed at day 0. (**A**) G6 VP7 IgG levels, (**B**) P[5] VP4* IgG levels, (**C**) G6 VP7 IgA levels, (**D**) P[5] VP4* IgA levels. Horizontal axis represents multiple sample collection time-points. Vertical axis represents geometric mean antibody titers (* *p* < 0.3; ** *p* < 0.002; *** *p* < 0.001).

Colostrum and milk samples were collected at farrowing (day 0) and then at weekly interval until weaning (days 7, 14 and 21). At day 0, treatment group 1 and 2 had significantly higher G6 IgG Ab titers compared to group 3 (1 NPE) and numerically higher IgG levels than the control group (Figure 4A). Group 1 P[5] IgG Ab titers at day 0 were significantly higher compared to groups 2, 3 and the control group (Figure 4B). Group 2 and 3 also had significantly higher IgG GMT levels than the control group at day 0 (Figure 4B). Overall at day 0, group 1 had significantly higher colostrum IgG titers for both antigens compared to the treatment groups, and either significantly or numerically higher IgG titers than the control group (Figure 4A,B). As expected, the colostrum IgG levels for both antigens were highest at day 0, which rapidly declined and reached the baseline at day 7, and remained so during the subsequent sampling points (Figure 4A,B).

At day 0, G6 IgA levels were numerically higher compared to other groups (Figure 4C). In contrast, colostrum P[5] IgA titers were significantly higher for group 1 compared to group 3 and control group and numerically higher than group 2 (Figure 4D). Overall, IgA titers declined at day 7 and then gradually increased until weaning (Figure 4C,D). Group 2, 3 and control IgA titers for both proteins at weaning matched or exceeded their titers in colostrum at day 0. However, treatment group 1 IgA titers for both antigens did not reach the colostrum IgA levels at day 0 (Figure 4C,D). The IgG and IgA VP4* (P[5]) titers were manifold higher than IgG and IgA VP7 (G6) titers (Figure 4A–D).

#### 3.2.3. Piglet Serum

**Figure 5:**
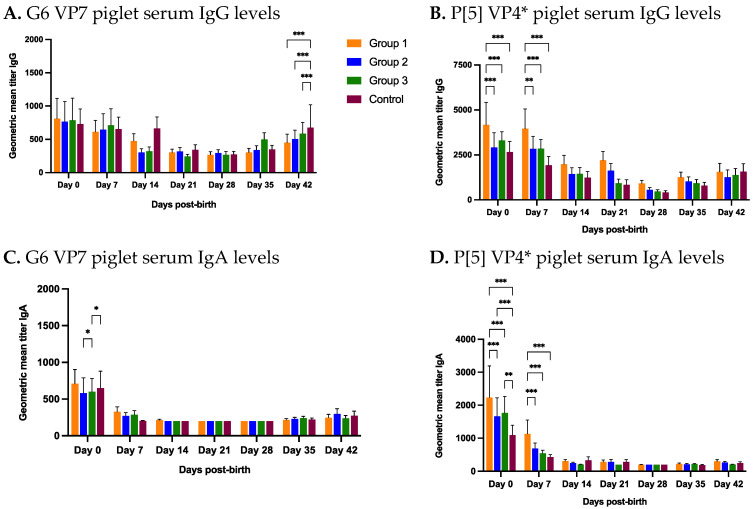
Longitudinal piglet serum antibody levels against RVC. Progression of RVC IgG and IgA levels over time in piglet serum born to gilts receiving three (group 1), two (group 2), one (group 3) or no (group 4) doses of natural planned exposure. Gilts farrowed at day 0. (**A**) G6 VP7 IgG levels, (**B**) P[5] VP4* IgG levels, (**C**) G6 VP7 IgA levels, (**D**) P[5] VP4* IgA levels. Horizontal axis represents multiple sample collection time-points. Vertical axis represents geometric mean antibody titers (* *p* < 0.3; ** *p* < 0.002; *** *p* < 0.001).

Piglet serum samples were collected at birth and then at weekly interval until 6 weeks of age (day 42). At birth (day 0), none of the study groups had significantly different G6 IgG levels, which ranged from 729.38 (lowest) for control group to 810.76 (highest) for group 1 (Figure 5A). However, day 0 group 1 P[5] IgG levels were significantly higher (GMT 4177.68) than group 2 (GMT 2914.92), 3 (GMT 3307.38) and control (GMT 2527.28) (Figure 5B). P[5] IgG levels of group 1 remained significantly higher than other groups at day 7 (Figure 5B). G6 IgG levels followed a gradual decrease until day 28 (Figure 5A). An increase in G6 IgG titers for all study groups was observed days 35 and 42 of piglets’ age (Figure 5A). At day 42, group 4 (control) showed significantly higher serum IgG levels compared to the group 1, 2 and 3 (Figure 5A). P[5] IgG titers for all groups declined post-birth reaching the baseline (serum dilution 1:200) at day 28 (Figure 5B). Similar to G6 IgG levels, an increase in P[5] IgG levels was observed at days 35 and 42 of sample collection (Figure 5B).

At day 0, group 1 G6 IgA piglet serum levels were higher than all other groups although levels were not significantly different (Figure 5C). However, group 2 IgA levels were significantly different compared to group 3 and group 3 IgA levels were significantly different than control group at day 0. (Figure 5C). Overall, serum G6 IgA levels of all four groups were highest at day 0, which rapidly declined at day 7 and subsequently reached the baseline (1:200). Group 1 P[5] IgA levels were significantly higher (GMT 2235.38) than group 2 (GMT 1665.28), 3 (GMT 1766.54) and control group (GMT 1090.19) at day 0 (Figure 5D). Interestingly, group 1 P[5] IgA levels remained significantly higher than other study groups at day 7 (Figure 5D). G6 and P[5] serum IgA levels showed minimal increase post-weaning compared to serum IgG levels (Figure 5A–D).

### 3.3. Levels of Antibodies against RVC Were Lower Than RVA in Colostrum

**Figure 6:**
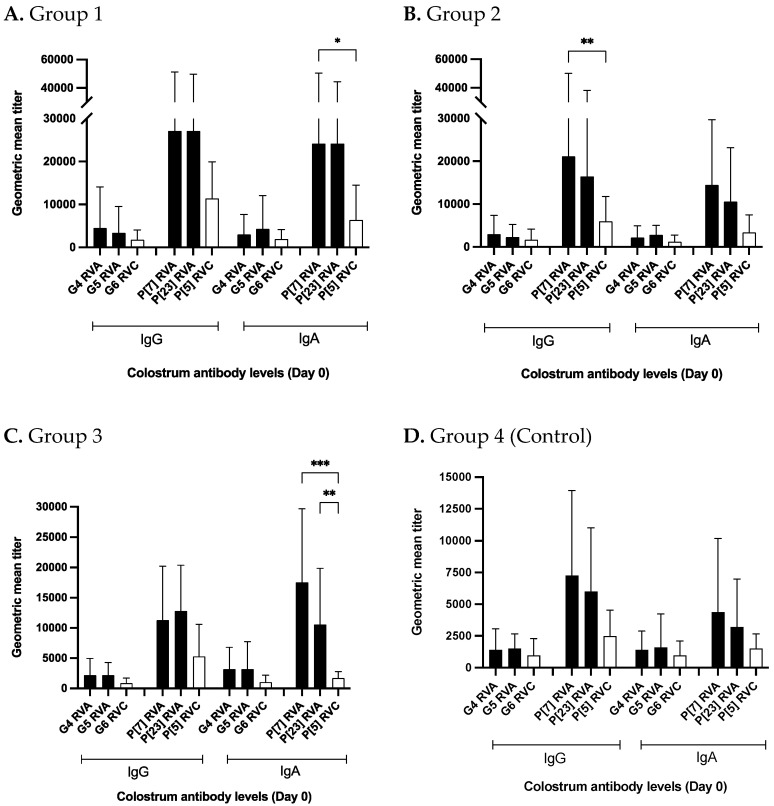
Groupwise comparison of antibody levels against RVA and RVC in colostrum (day 0). Vertical axis represents geometric mean antibody titers for respective study groups. Horizontal axis represent IgG and IgA levels against RVA and RVC genotypes. Black shaded bars represent different RVA genotypes and white colored bars represent RVC genotypes. (**A**) RVA and RVC antibody levels in group 1, (**B**) RVA and RVC antibody levels in group 2, (**C**) RVA and RVC antibody levels in group 3, and (**D**) RVA and RVC antibody levels in group 4 (Control group). Significance levels (*p*-values) only describing comparison between RVA and RVC genotypes are illustrated (* *p* < 0.3; ** *p* < 0.002; *** *p* < 0.001).

The NPE material fed to the study gilts also contained RVA strains. The results of antibody response to RVA strains (G4, G5, P[7] and P[23]) in sow colostrum/milk have been discussed elsewhere [34]. In this section, day 0 colostrum RVA and RVC antibody levels are summarized. For all 4 study groups, colostrum RVC IgG and IgA titers were lower than RVA titers irrespective of the G and P-type (Figure 6A–D). G4/G5 RVA IgG and IgA levels for all 4 groups were numerically higher than G6 RVC levels (Figure 6A–D). P[7] and P[23] RVA antibody levels were either significantly or numerically higher than P[5] RVC antibody levels (Figure 6A–D). For group 1, P[7] and P[23] RVA IgA antibody levels were significantly higher than P[5] RVC IgA levels (Figure 6A). In group 2, P[7] RVA IgG levels were significantly higher compared to P[5] RVC IgG levels (Figure 6B). Group 3 had significantly higher P[7] and P[23] RVA IgA and numerically higher IgG levels than P[5] RVC (Figure 6C). Control group P-type RVA IgG and IgA levels were always numerically higher than P[5] RVC (Figure 6D). VP4* (P-specific) IgG and IgA titers were manifold higher than VP7 (G-specific) antibody titers for all 4 groups (Figure 6A–D).

### 3.4. Piglet Serum at Birth Has Lower Antibodies against RVC Than RVA

**Figure 7:**
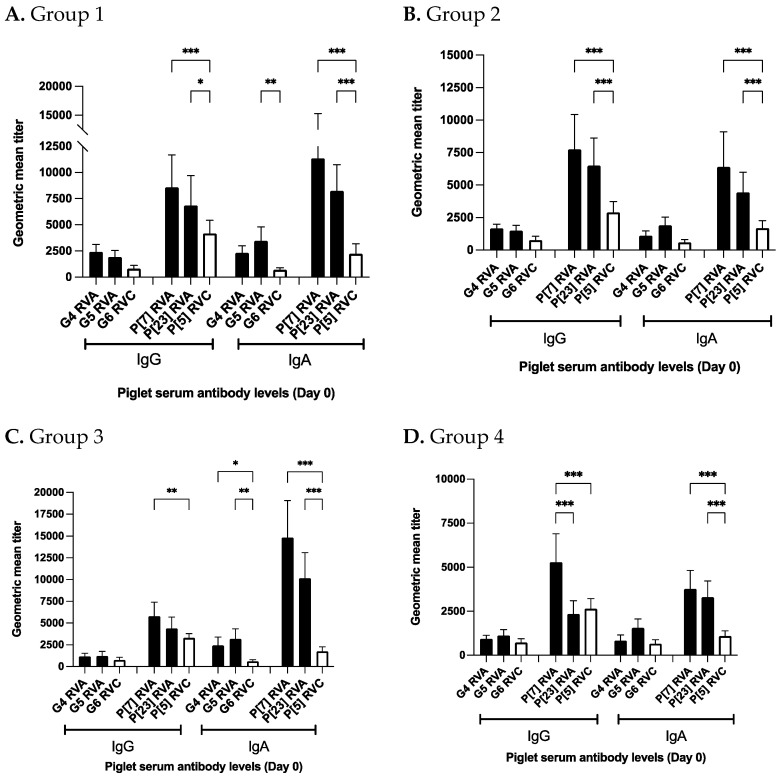
Groupwise comparison of antibody levels against RVA and RVC in day 0 piglet serum samples. Vertical axis represents geometric mean antibody titers for respective study groups. Horizontal axis represent IgG and IgA levels against RVA and RVC genotypes. Black shaded bars represent different RVA genotypes and white colored bars represent RVC genotypes. (**A**) RVA and RVC antibody levels in group 1, (**B**) RVA and RVC antibody levels in group 2, (**C**) RVA and RVC antibody levels in group 3, and (**D**) RVA and RVC antibody levels in group 4 (Control group). Significance levels (*p*-values) only describing comparison between RVA and RVC genotypes are illustrated (* *p* < 0.3; ** *p* < 0.002; *** *p* < 0.001).

Genotype-specific RVA (G4, G5, P[7] and P[23]) antibody levels for piglet serum have been discussed elsewhere [34]. For all groups, day 0 piglet serum RVC IgG and IgA titers were lower than RVA titers except for higher P[5] RVC IgG levels than P[23] RVA in the control group (Figure 7A–D). For group 1 and 2, day 0 P[7] and P[23] RVA IgG and IgA levels were significantly higher compared to P[5] RVC antibody levels (Figure 7A,B). G4 and G5 RVA antibody levels were either significantly or numerically higher than G6 RVC antibody levels (Figure 7A–D). Importantly, control group P[7] and P[23] RVA IgA levels were significantly higher compared to P[5] RVC IgA levels (Figure 7D). P[7] RVA IgG levels were also significantly higher than P[5] RVC IgA levels. No significant difference was observed between P[23] RVA and P[5] RVC IgG titers (Figure 7D).

### 3.5. RVC Fecal Shedding in Piglets and Antibody Response

NPE material fed to the gilts contained both RVA and RVC strains. Realtime PCR of feedback (NPE) material revealed RVC ct-values of 32.55, 29.32 and 30.30 for feedback 1 (5 WPF), 2 (4 WPF) and 3 (3 WPF), respectively. In contrast, low RVA Ct-values of 24.42, 22.46 and 24.15 were detected for feedback 1 (5 WPF), 2 (4 WPF) and 3 (3 WPF). Gilt and piglet RVC fecal shedding results have been described in detail earlier and also summarized in the supplementary Table S1 [29]. As expected, piglets’ fecal swabs collected within 24 h of farrowing were negative for RVC by qRT-PCR. RVC was first shed at day 7 in all 4 groups, but high viral load of RVC (low ct-values) were only observed in the control group piglets (Supplementary Table S1). Multiple litters in all 4 groups shed RVC before weaning (prior to day 21). At day 7, when RVC was first detected, the piglet pools from control gilts contained the most RVC positive litters (58%), while 17%, 42% and 9% of litters in groups 1, 2, and 3 were positive, respectively. In group 4 (control), higher number of RVC positive litters and high viral load at day 7 correlated with low colostrum IgA G6 and P[5] IgA colostrum IgA levels. Although, day 0 G6 RVC piglet serum IgA levels for all 4 groups were in a close range (Figure 5C), control group had lowest P[5] IA titers in piglet serum compared to the 3 treatment groups (Figure 5D). Day 7 piglet serum antibody levels revealed that litter P[5] IgA titers lower than 800 significantly correlated with litter being RVC positive.

### 3.6. Sequence Analysis of RVC from NPE and Piglet Feces

A total of 30 pre-weaning piglet fecal samples representing pre-weaning RVC shedding by all 4 groups were sequenced to investigate genetic changes in response to lactogenic immunity. Complete RVC genome could only be recovered from 11 fecal samples representing treatment group 2, 3 and control group piglets (Table 2). Sequencing revealed a RVC genome constellation of G6-P[5]-I5-R1-C1-M1-A1-N6-Tu-Eu-H1 from all 11 samples regardless of the shedding week (1, 2 or 3). G and P-type combination (G6P[5]) detected in these 11 samples was similar to the RVC genotypes present (G6, P[5]) in the original NPE material fed to the gilts. Sequence analysis revealed very high nucleotide (98.62–99.90%) and amino acid (98.22–100%) percent identity among 11 G6 VP7 sequences from piglet feces and parent G6 NPE strain. Six G6 sequences completely matched the G6 NPE strain. To determine the sequence variation, the neutralizing epitopes of the 11 G6 sequence recovered from piglet feces were compared to the parent G6 strain. Five G6 sequences illustrated deletions at 245–247 amino acid positions and also differed with the parent G6 strain at one amino acid position 248 (L248I) (Table 3). Other 6 G6 sequences from piglets completely matched the parent G6 strain. Similarly, P[5] VP4 sequences from piglet feces shared very high nucleotide (99.09–100%) and amino acid (99.11%) percent identity with parent P[5] strain in the NPE material. VP4 sequences from piglets differed with parent P[5] RVC strain at amino acid positions 41 (T41I), 203 (I203L), 262 (R262W) and 350 (G350D).

## 4. Discussion

Presently, NPE is the only cost-effective method of stimulating passive lactogenic immunity to protect piglets against RVCs. However, due to lack of serological tools to detect RVC antibodies, effective of NPE protocols has not been evaluated. To fill this knowledge gap, we optimized VP7 (G6) and truncated VP4* (P[5]) specific indirect ELISAs and investigated antibody responses against RVC in gilts after NPE and passive immunity in their piglets. To our knowledge, this is the first longitudinal study to investigate antibody levels against RVC in gilts/sows (pre and post-farrow serum), colostrum/milk, and their piglets at multiple time-points.

In gilt serum, 2 doses of NPE in group 1 and 1 dose of NPE in groups 2 and 3 resulted in increased IgG and IgA levels at 3 WPF reflecting the development of active immunity against RVC in gilts. Antibody levels dropped at farrowing (F) suggesting transport of RVC-specific antibodies into the colostrum. Drop in gilt serum IgG levels was more distinct compare to serum IgA levels (Figure 2A–D). Similar trend has been observed for RVA antibody levels at farrowing [34]. Possible reasons for this difference could be the release of IgA synthesized in mammary parenchyma into the gilt/sow serum or reduced transportation of serum IgA into exocrine fluid [36]. A study found increased sow serum IgA levels against RVA during last weeks of gestation in contrast to serum IgG levels, which dropped sharply at farrowing [36]. However, the difference between serum IgG and IgA levels at farrowing may not truly indicate their respective levels in colostrum, as only 24–54% of IgA in colostrum comes from serum whereas all colostral IgG is derived from serum in swine [37].

Since no intra-uterine passage of immunoglobulins occurs in swine during gestation, piglets are born agammaglobulinemic and uptake of pathogen-specific colostrum/milk immunoglobulins is critical for their survival [26,38,39]. We found that group 1 has significantly or numerically higher colostral (day 0) IgG and IgA titers compared to other groups for both antigens, suggesting that 3 doses of NPE administered to group 1 gilts prior to farrowing was able to better stimulate maternal immunity compared to other NPE doses. Moreover, control group gilts (no NPE) had the lowest colostral antibody levels resulting in highest RVC fecal shedding (58%) in piglets at day 7 of age compared to 17%, 42% and 9% in groups 1, 2 and 3, respectively. Rapid drop in the colostrum IgG levels in day 7 milk occurred in parallel with rapid rise in sow serum IgG titers post-farrowing until weaning. On the contrary, IgA levels in milk increased steadily post day 7 until day 21, suggesting the increased local production of RVC-specific IgA in mammary glands and subsequent secretion in the milk. Similar trends of colostrum/milk IgG and IgA have been observed against multiple genotypes of RVA post-farrowing [34]. For example, group 1 gilts with 3 doses of NPE had higher IgG and IgA levels for both RVC and RVA compared to other treatment groups.

Data regarding lactogenic protection against porcine RVC is very rare and most of the swine RV lactogenic immunity studies have been done for RVA [30,40]. Recently, RVC antibody titers in gilt/sow milk and serum samples were reported using genotype-specific and cocktail of genotype-specific virus like particles (VLPs) based indirect ELISAs [26]. Authors reported no difference in levels of IgG and IgA against RVC G6 genotype in milk collected after 2–11 days of farrowing. Similar to Chepngeno study, we also observed that control group gilts (no NPE) had similar G6 RVC-specific IgG and IgA levels in day 0 colostrum samples. Comparison of antibody titers against RVA and RVC in colostrum (day 0) revealed that for all 4 study groups, anti-RVC antibody titers were lower (significantly or numerically) compared to RVA titers irrespective of the G and P-type. Variation in antibody levels to RVA and RVC in colostrum could be due to differences in virus replication in gilts as evidenced by RV gilt fecal shedding results before farrowing [29]. For RVA, 1st dose of NPE in treatment groups resulted in 71.4% (25/35) gilts shedding RVA at 4.5 weeks pre-farrow in comparison to only 20% (7/35) gilts shedding RVC after 1st of NPE [29]. Overall, NPE administration in treatment groups resulted in higher levels of RVA shedding in gilts compared to RVC.

Significantly higher group 1 P[5] antibody levels in day 0 and 7 piglet serum mirrored higher P[5] IgA levels of group 1 in colostrum suggesting that 3 doses of NPE stimulated slightly enhanced antibody response against RVC than other NPE doses. However, G6 IgG levels in day 0 piglet serum were not significantly different for any group and G6 IgA levels were in a narrow range for all groups. Significant differences in antibody levels were also observed against RVA and RVC in day 0 piglet serum (Figure 7A–D). Importantly, RVC IgA titers in day 0 piglet serum were significantly lower than RVA titers irrespective of G and P-type (Figure 7A–D). We hypothesize that higher ct-values of RVC (ct-values 32.55, 29.32 and 30.30) compared to RVA (ct 24.43, 22.46 and 24.15) in the NPE material could not induce sufficient immunity and resulted in low colostrum RVC antibody levels. Low anti-RVC antibody levels failed to passively protect piglets from natural RVC infection in the farrowing room. RV fecal shedding data from piglets also support this assumption. Only two litters (5.8%, 2/34) shed RVA prior to weaning compared to 8 litters (23.5%, 8/34) at day 7 and 18 litters (53%, 18/34) each at day 14 and 21 for RVC (supplementary Table S1) [34]. Very less RVA shedding in the farrowing room suggests that the better passive immunity was induced against RVA, which had the lower ct-values in the NPE compared to RVC.

Low RVC antibody titers generated in gilts are known to be associated with higher rates of clinical disease in piglets [26]. We also found that in piglet litter with P[5] IgA GMTs less than 800 were positively correlated with litter being tested RVC positive. Interestingly, we observed that day 0 piglet serum P[7] and P[23] RVA IgA levels in the control group were significantly higher compared to P[5] RVC IgA levels (Figure 7D). This finding is significant because control group gilts did not receive pre-farrow NPE. Higher anti-RVA IgA levels in control group piglets at birth suggest higher passive immunity to RVA than RVC in the absence of NPE administration to gilts. This finding reflects the dynamics of RVA and RVC antibody response in gilts and piglets in the absence of NPE.

NGS detected G and P-type combination of G6P[5] in pre-weaning piglet fecal samples which was similar to the RVC genotypes present (G6, P[5]) in the original NPE material fed to the gilts. Moreover, high nucleotide and amino acid percent identities and very few point mutations among RVC strains from piglets reiterate that lactogenic immunity stimulated by the RVC NPE was not sufficient to prevent piglets from RVC infections in the farrowing room. In contrast, we earlier identified a G and P-type RVA combination (G11P[34]) in two pre-weaning samples shedding RVA, which was different from the genotypes present (G4, G5, P[7] and P[23]) in the original NPE material fed to the gilts [34]. The VP7 and VP4 proteins of RVs independently elicit neutralizing and protective antibody response [41]. We observed that RVC IgG and IgA levels induced by VP4* were higher compared to VP7, which is possibly due to presence of more neutralizing epitopes on VP4 compared to VP7. We have earlier observed similarly high levels of antibodies against VP4* than VP7 for RVA [34].

In summary, treatment group one with 3 doses of pre-farrow NPE resulted in significantly higher anti-RVC antibody levels in colostrum. Although 3 doses of NPE appear better in stimulating lactogenic immunity, none of the NPE doses were able to prevent RVC shedding by piglets in the farrowing room, reflecting low RVC load (higher ct values) in the NPE material. Our results suggest that gilt colostrum and piglet serum contain significantly lower levels of antibodies to RVC than RVA, which possibly explains higher prevalence of RVC in neonatal piglets. Since RVA is more prevalent in swine farms and gilts normally carry higher levels of antibodies against RVA than RVC, it is proposed to administer only RVC NPE (no RVA) to gilts at 5,4, and 3 weeks before farrowing. Furthermore, more research is required to find methods to increase RVC viral load in the NPE material. Results of this study expand our understating of the antibody response to RVC in swine and the role of NPE in providing lactogenic immunity to naïve piglets.

## Figures and Tables

**Table 1:** Feedback Administration and sample collection (serum, colostrum and milk) schedule. Gilts and five piglets per gilt were sampled individually.

		−5W	−4W	−3W	0W (Farrowing)	1W	2W	3W (Weaning)	4W	5W	6W
**Group 1**	**Gilts (n = 12)**	X *	*	X *	X+	+	+	X+			
	5 piglets/gilt				X	X	X	X	X	X	X
**Group 2**	**Gilts (n = 12)**	X *		X *	X+	+	+	X+			
	5 piglets/gilt				X	X	X	X	X	X	X
**Group 3**	**Gilts (n = 11)**	X *		X	X+	+	+	X+			
	5 piglets/gilt				X	X	X	X	X	X	X
**Group 4**	**Gilts (n = 11)**	X		X	X+	+	+	X+			
	5 piglets/gilt				X	X	X	X	X	X	X

X = gilt serum collection, X = piglet serum collection, (+) = colostrum and milk collection, and * = NPE administration.

**Table 2:** Genome constellation of RVC strains detected in piglet feces.

Litter ID	Group	Week	RVC Ct	Genome Constellation
40960	2	1	17.66	G6-P[5]-I5-R1-C1-M1-A1-N6-Tx-Ex-H1
		2	18.39	G6-P[5]-I5-R1-C1-M1-A1- N6-Tx-Ex-H1
41045	2	2	22.57	G6-P[5]-I5-R1-C1-M1-A1- N6-Tx-Ex-H1
		3	21.7	G6-P[5]-I5-R1-C1-M1-A1- N6-Tx-Ex-H1
41009	3	2	17.48	G6-P[5]-I5-R1-C1-M1-A1- N6-Tx-Ex-H1
		3	19.68	G6-P[5]-I5-R1-C1-M1-A1- N6-Tx-Ex-H1
41285	3	2	19.64	G6-P[5]-I5-R1-C1-M1-A1- N6-Tx-Ex-H1
		3	22.49	G6-P[5]-I5-R1-C1-M1-A1- N6-Tx-Ex-H1
41014	4	1	17.94	G6-P[5]-I5-R1-C1-M1-A1- N6-Tx-Ex-H1
41174	4	1	17	G6-P[5]-I5-R1-C1-M1-A1- N6-Tx-Ex-H1
41025	4	2	21.13	G6-P[5]-I5-R1-C1-M1-A1- N6-Tx-Ex-H1

**Table 3:** Antigenic variation in the VP7 protein among the RVC strains recovered from piglet feces and parent NPE RVC strain (G6).

	Amino Acid Positions in VP7																								
	84	88	89	90	91	92	150	151	152	153	154	155	156	194	195	197	226	245	246	247	248	249	250	251	252
G6-Parent Sequence	A	S	P	G	P	G	E	P	K	N	S	E	A	E	D	D	D	S	S	S	L	N	Q	L	Q
RVC/PIG/USA/S8/2017/G6	**.**	**.**	**.**	**.**	**.**	**.**	**.**	**.**	**.**	**.**	**.**	**.**	**.**	**.**	**.**	**.**	**.**	**.**	**.**	**.**	**.**	**.**	**.**	**.**	**.**
RVC/PIG/USA/S18/2017/G6	**.**	**.**	**.**	**.**	**.**	**.**	**.**	**.**	**.**	**.**	**.**	**.**	**.**	**.**	**.**	**.**	**.**	**.**	**.**	**.**	**.**	**.**	**.**	**.**	**.**
RVC/PIG/USA/S20/2017/G6	**.**	**.**	**.**	**.**	**.**	**.**	**.**	**.**	**.**	**.**	**.**	**.**	**.**	**.**	**.**	**.**	**.**	--	--	--	I	**.**	**.**	**.**	**.**
RVC/PIG/USA/S31/2017/G6	**.**	**.**	**.**	**.**	**.**	**.**	**.**	**.**	**.**	**.**	**.**	**.**	**.**	**.**	**.**	**.**	**.**	--	--	--	I	**.**	**.**	**.**	**.**
RVC/PIG/USA/S21/2017/G6	**.**	**.**	**.**	**.**	**.**	**.**	**.**	**.**	**.**	**.**	**.**	**.**	**.**	**.**	**.**	**.**	**.**	**.**	**.**	**.**	**.**	**.**	**.**	**.**	**.**
RVC/PIG/USA/S32/2017/G6	**.**	**.**	**.**	**.**	**.**	**.**	**.**	**.**	**.**	**.**	**.**	**.**	**.**	**.**	**.**	**.**	**.**	--	--	--	I	**.**	**.**	**.**	**.**
RVC/PIG/USA/S22/2017/G6	**.**	**.**	**.**	**.**	**.**	**.**	**.**	**.**	**.**	**.**	**.**	**.**	**.**	**.**	**.**	**.**	**.**	**.**	**.**	**.**	**.**	**.**	**.**	**.**	**.**
RVC/PIG/USA/S33/2017/G6	**.**	**.**	**.**	**.**	**.**	**.**	**.**	**.**	**.**	**.**	**.**	**.**	**.**	**.**	**.**	**.**	**.**	**.**	**.**	**.**	**.**	**.**	**.**	**.**	**.**
RVC/PIG/USA/S12/2017/G6	**.**	**.**	**.**	**.**	**.**	**.**	**.**	**.**	**.**	**.**	**.**	**.**	**.**	**.**	**.**	**.**	**.**	--	--	--	I	**.**	**.**	**.**	**.**
RVC/PIG/USA/S13/2017/G6	**.**	**.**	**.**	**.**	**.**	**.**	**.**	**.**	**.**	**.**	**.**	**.**	**.**	**.**	**.**	**.**	**.**	**.**	**.**	**.**	**.**	**.**	**.**	**.**	**.**
RVC/PIG/USA/S24/2017/G6	**.**	**.**	**.**	**.**	**.**	**.**	**.**	**.**	**.**	**.**	**.**	**.**	**.**	**.**	**.**	**.**	**.**	**.**	**.**	**.**	**.**	**.**	**.**	**.**	**.**

## Data Availability

The raw data supporting the conclusions of this article will be made available by the authors, without undue reservation.

## Supplementary Materials

The following are available online at https://www.mdpi.com/article/10.3390/v14102250/s1, Table S1: Longitudinal RVC detection levels (Ct-values) in piglets’ feces (5 piglets/litter) at multiple time points.
