# Supplementary material for: Antibody Response to Rotavirus C Pre-Farrow Natural Planned Exposure to Gilts and Their Piglets
Source: Viruses. 2022 Oct 14;14(10):2250. doi: 10.3390/v14102250 (PMC9610825; doi:10.3390/v14102250)
Supplement: Supplementary file 1 [file viruses-14-02250-s001.zip › viruses-1905171-supplementary.pdf]

Supplementary table S1: Longitudinal RVC detection levels (Ct-values) in piglets' feces (5 piglets/litter) at multiple time points

	Sow ID	RVC detected (Ct values) in piglet feces at different time points						
		Day 0	Day 7	Day 14	Day 21	Day 28	Day 35	Day 42
Group 1	40399					24.14	20.86	27.56
	38868					25.27	20.28	26.19
	41031		24.29	20.43	25.59	27.50	19.37	26.00
	41071		25.78	25.18	25.23	19.15	19.65	22.98
	41049			31.06		17.29	18.36	27.75
	41267			25.05	35.44	24.63	19.61	25.15
	40973			28.77		32.01	18.20	26.73
	40652				29.86	24.27	18.35	22.11
	40956					29.02	17.37	19.37
	41262			25.43	30.59	20.27	18.31	23.25
	41144					20.01	17.73	17.02
	41181					27.05	18.03	24.98
Group 2	41289					23.19	17.51	17.10
	41030		33.79	23.75	27.22	33.83	19.23	20.90
	41052				27.10	28.58	29.18	26.35
	40960		23.71	20.96	39.43	27.29	18.55	25.11
	41011			23.96	27.45	20.81	24.97	24.43
	41010		28.99	23.43	29.14	33.01	17.35	25.37
	40984		22.64	25.16	23.09	30.85	15.72	22.16
	41069					28.43	19.13	26.00
	41046					20.83	17.11	25.07
	40954					22.85	17.53	18.47
	41045		23.38	24.82	25.22	26.01	20.09	23.23
Group 3	41009			20.23	23.09	25.01	18.19	26.85
	41027			22.72	24.43	20.06	19.18	26.51
	40975			20.53	26.01	26.07	23.45	26.26
	40995			22.32	26.43	20.05	19.92	22.30
	41004			21.05	24.05	29.13	20.20	24.16
	40994					24.89	17.78	18.70
	41062					23.16	19.20	21.53
	41249					23.41	15.91	24.98
	41285		22.23	21.95	24.04	26.69	26.51	26.88
	40996					24.10	22.41	23.20
	41219					24.40	20.45	23.07
Group 4	40969			34.91		23.45	18.55	29.13

	41112					24.43	20.81	21.38
	41025			21.48	28.71	28.38	18.73	24.57
	41014		19.48	18.75	28.88	31.44	30.51	21.57
	41053		28.06	23.33	23.63	28.62	20.23	20.83
	41070		25.84	34.54		24.47	23.07	19.12
	41174		17.19	29.79	29.80	30.27	21.81	28.31
	40964		19.34	29.19	35.01	33.39	22.35	27.36
	40979		20.88	26.62	30.11	28.17	18.84	32.10
	41026						19.15	24.84
	41057		19.86	28.67	34.14	27.87	18.18	24.31
